# Grazing exclusion by fencing non-linearly restored the degraded alpine grasslands on the Tibetan Plateau

**DOI:** 10.1038/s41598-017-15530-2

**Published:** 2017-11-09

**Authors:** Jianshuang Wu, Yunfei Feng, Xianzhou Zhang, Susanne Wurst, Britta Tietjen, Paolo Tarolli, Chunqiao Song

**Affiliations:** 1 Lhasa Plateau Ecosystem Research Station, Key Laboratory of Ecosystem Network Observation and Modelling, Institute of Geographic Sciences and Natural Resources Research, Chinese Academy of Sciences, Beijing, 100101 China; 2 Freie Universität Berlin, Institute of Biology, Functional Biodiversity, Königin-Luise-Straße 1-3, 14195 Berlin, Germany; 3 Freie Universität Berlin, Institute of Biology, Biodiversity/Theoretical Ecology, Altensteinstraße 34, 14195 Berlin, Germany; 4 Department of Land, Environment, Agriculture and Forestry, University of Padova, Agripolis, viale dell’Università 16, Legnaro (PD), Italy; 5 Department of Geography, University of California, Los Angeles, CA 90095 USA

## Abstract

Resilience is an important aspect of the non-linear restoration of disturbed ecosystems. Fenced grassland patches on the northern Tibetan Plateau can be used to examine the resistance and resilience of degraded alpine grasslands to grazing and to a changing climate. To examine the non-linearity of restoration, we used moderate resolution imaging spectroradiometer (MODIS) normalized difference vegetation index (NDVI) as a proxy for productivity during a ten-year restoration by fencing. Degraded alpine grasslands exhibited three restoration trajectories: an equilibrium in meadows, a non-linear increase across steppes, and an abrupt impulse in desert-steppes following a slight increase in productivity. Combined with weather conditions, the ten-year grazing exclusion has successfully enhanced the NDVI on the most degraded steppes, but did not do so efficiently on either meadows or desert-steppes. Warming favors the NDVI enhancement of degraded meadows, but higher temperatures limited the restoration of degraded steppes and desert-steppes. Precipitation is necessary to restore degraded alpine grasslands, but more precipitation might be useless for meadows due to lower temperatures and for desert-steppes due to limitations caused by the small species pool. We suggest that detailed field observations of community compositional changes are necessary to better understand the mechanisms behind such non-linear ecological restorations.

## Introduction

Environmental change on the Tibetan Plateau is receiving increasing attention due to the ecological consequences of changing climate^1–7^. This plateau plays an important role in ecological security by safeguarding both the environment and the economy of mainland China^8–10^. As Asia’s water tower, the Tibetan Plateau provides fresh water to several billions of residents in the local and surrounding regions^11–13^. The Tibetan Plateau is known as the “Third Pole of the Earth”, not only due to its severe physical environments but also because the alpine ecosystems there are as fragile as those in the Arctic and Antarctic^1,8,14^. Grasslands on the Tibetan Plateau are important for the welfare of humans and wildlife^12,15^, however, the relative contributions of climate warming and human activities to ecosystem functionality change are not fully understood.

Tibetan alpine grasslands are sensitive and vulnerable to climate change and human disturbance^4–6,16–18^. These ecosystems have experienced persistent overgrazing and an accelerated warming in recent decades. In 2010, the livestock population in Tibet increased to nearly double the estimated capacity of all available pastures^19^. Furthermore, the warming rate of the Tibetan Plateau is faster than that of the East China and the Northern Hemisphere and even double compared to the global average^14,15^. Overgrazing and warming mainly contribute to the degradation of natural alpine grasslands, leading to productivity declines, land desertification, and an increase in noxious weeds^20^. Hence, the restoration of degraded alpine grasslands has become one of the most critically urgent issues for the development of Tibet^19^.

The Chinese government expects to effectively recover degraded montane and alpine grasslands; therefore, metal fences for grazing exclusion seasonally or year-round were constructed to assist in the self-restoration of degraded pastures on the Tibetan Plateau^21–23^. The total area of fenced grassland patches is approximately 4.75 million hectares on the northern Tibetan Plateau (Supplementary Figure S1 and Table S1), accounting for 10.0% of available alpine pastures. Thus, examining the effectiveness of grazing exclusion by fencing and understanding the mechanisms behind ecosystem self-restoration becomes a great challenge and will inform grassland conservation and sustainable management in the future.

Recent studies have debated on how grazing exclusion affects plant diversity^24–26^, biomass production^27,28^, and soil nutrients^29–32^. Grazing exclusion has been found to only favor certain plant functional groups, such as grasses and sedges in alpine meadows^23^. Short-term grazing exclusion can enhance aboveground biomass relative to grasslands under grazing^25,28,29^, but cannot significantly alter species assembly^24,25,33,34^. Moreover, a short-term (less than five years) grazing exclusion duration (GED) has no impact on the soil nutrients of alpine steppes and desert-steppes^32^. Overall, the mechanisms underlying the self-restoration of degraded pastures in response to grazing exclusion remain unclear.

**Figure 1:**
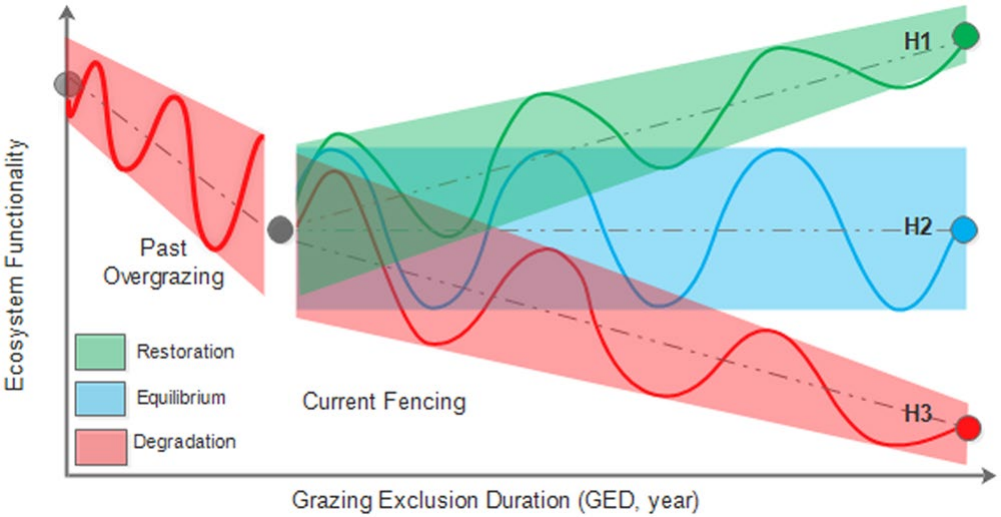
Three hypothetical trajectories proposed to describe the non-linear self-restoration scenarios of degraded alpine grasslands on the northern Tibetan Plateau.

Ecological resilience is an important concept for understanding the non-linear dynamics observed in diverse ecosystems^35,36^. Resilience is generally described as the amount of disturbance that a system can tolerate without interior changes, or as the time for ecosystems to return a stable state following a perturbation or degradation^37,38^. Theories predict that ecosystem restoration likely goes through a non-linear trajectory, through which the tendency and variability over time can describe the direction and resilience of a restoring ecosystem, respectively (Fig. 1). However, little is known about the ecosystem resilience of degraded alpine grasslands on the Tibetan Plateau, especially with respect to non-linear self-restoration driven by climatic and human influences^39,40^.

The normalized difference vegetation index (NDVI) from moderate resolution imaging spectroradiometer (MODIS) data can well reflect the spatiotemporal patterns of vegetation activities over the Tibetan Plateau^41–44^. Verbesselt, *et al*.^45^ recently suggested that the NDVI can be used to monitor the resilience of tropical forests. Here, we used the MODIS NDVI as a proxy for ecosystem productivity (Supplementary Figure S2) to distinguish the relative importance of year-to-year weather conditions and grazing exclusion on the self-restoration of fenced degraded grasslands. We aim to answer, first, whether a ten-year grazing exclusion has successfully restored degraded grasslands and resulted in enhanced NDVI; second, whether the productivity of fenced degraded pastures of different grassland types was increasing; and finally, what are the relative contributions of weather conditions and grazing exclusion to the ecosystem self-restoration of these ecosystems.

We conceptualized three alternative trajectories to describe the non-linear self-restoration of degraded grasslands during a ten-year grazing exclusion by fencing (see Fig. 1). To test the following hypotheses, we took the average yearly NDVI before fencing (2001–2005) as the baseline and analyzed the yearly NDVI difference (ΔNDVI) of each fenced grassland patch after fencing from 2006 to 2015, relative to the baseline. The same procedure was also applied to growing season temperature (GST) and precipitation (GSP). The non-linearity and temporal variability in each variable, as well as in their relationships, were explored by using generalized linear models (GLMs) and generalized additive models (GAMs) (for details see Methods).

- H1. An increasing trend (green dashed linear line) with narrowing temporal variability (green solid line and shaded area) predicts grazing exclusion to be effective for promoting self-restoration;
- H2. An equilibrium state (blue solid non-linear line and shaded area) without any evident trend (blue dashed linear line) predicts grazing exclusion to be useless for grassland self-restoration;
- H3. An overall decreasing trend (red dashed linear line) with narrowing temporal variability (red solid non-linear line and shaded area) predicts grazing exclusion to effectively slow degradation.

## Results

**Figure 2:**
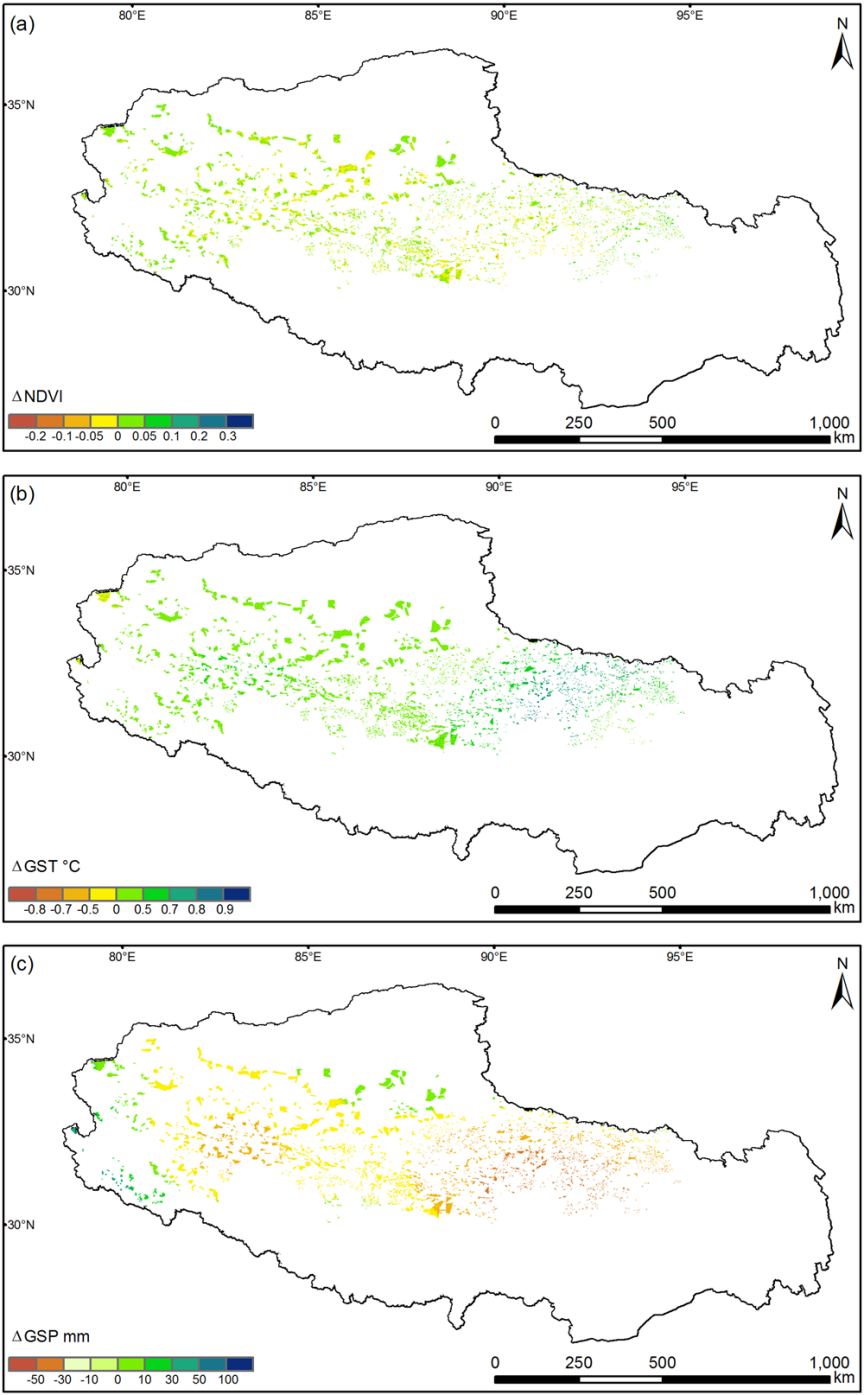
Changes in productivity and climate after fencing. Spatial distribution of the changes in **(a**) Normalized Difference Vegetation Index (ΔNDVI), (**b**) growing season temperature (ΔGST), and (**c**) growing season precipitation (ΔGSP) between the two sub-periods of before (2001–2005) and after fencing (2006–2015) for each fenced grassland patch on the northern Tibetan Plateau. This map was produced in ArcGIS 10.2 (http://www.esri.com).

Changes in the NDVI at the fenced patch scale vary among alpine grassland types on the northern Tibetan Plateau (Fig. 2 and Table 1). For 50.5% of all fenced grassland patches, the mean annual NDVI of 2006–2015 increased. However, for 39.6% and 9.9% of fenced grassland patches, the mean annual NDVI declined and showed no change between the two sub-periods of before and after being fenced, respectively.

**Table 1:** Percentage of grassland patch numbers where the NDVI and weather conditions between the two sub-periods of before (2001–2005) and after fencing (2006–2015) were increasing, decreasing, or no change within and across alpine grassland types on the northern Tibetan Plateau.

	NDVI			GST			GSP		
	increasing (%)	decreasing (%)	no change (%)	increasing (%)	decreasing (%)	no change (%)	increasing (%)	decreasing (%)	no change (%)
AM	54.12	42.01	3.87	99.64	0.17	0.17	0.66	98.02	1.32
AS	42.49	42.49	15.03	100.00	0.00	0.00	5.42	91.97	2.61
ADS	68.33	13.12	18.55	96.82	0.00	3.18	5.00	61.82	33.18
Total	50.51	39.63	9.87	99.55	0.08	0.37	99.55	0.08	0.37

AM, AS, and ADS stand for alpine meadow, steppe, and desert-steppe, respectively. GST and GSP are for growing season temperature and growing season precipitation, respectively. Mean variations of the after-fencing period from −0.01 to 0.01 for NDVI, from −0.05 to 0.05 °C for GST, and from −1 to 1 mm for GSP in relative to the average of the before-fencing period, are defined as no change.

The NDVI showed significant linear trends (*P* < 0.1), either increasing or decreasing (Supplementary Table S2), for only 6.5% of patches across the northern Tibetan Plateau. Linear regression failed to capture any directional signal of ecosystem self-restoration in fenced grassland patches (*P* > 0.1 in most patches, Supplementary Figure S3). Correlation analyses did not explain weather impacts on the NDVI variability over the ten-year fencing, either. The correlations of NDVI with weather conditions were low and nonsignificant for most patches (Supplementary Figure S4). For only 3.6% of the patches, the NDVI was found to be significantly correlated with GST, GSP, or both them at *P* < 0.1 (Supplementary Table S3).

**Figure 3:**
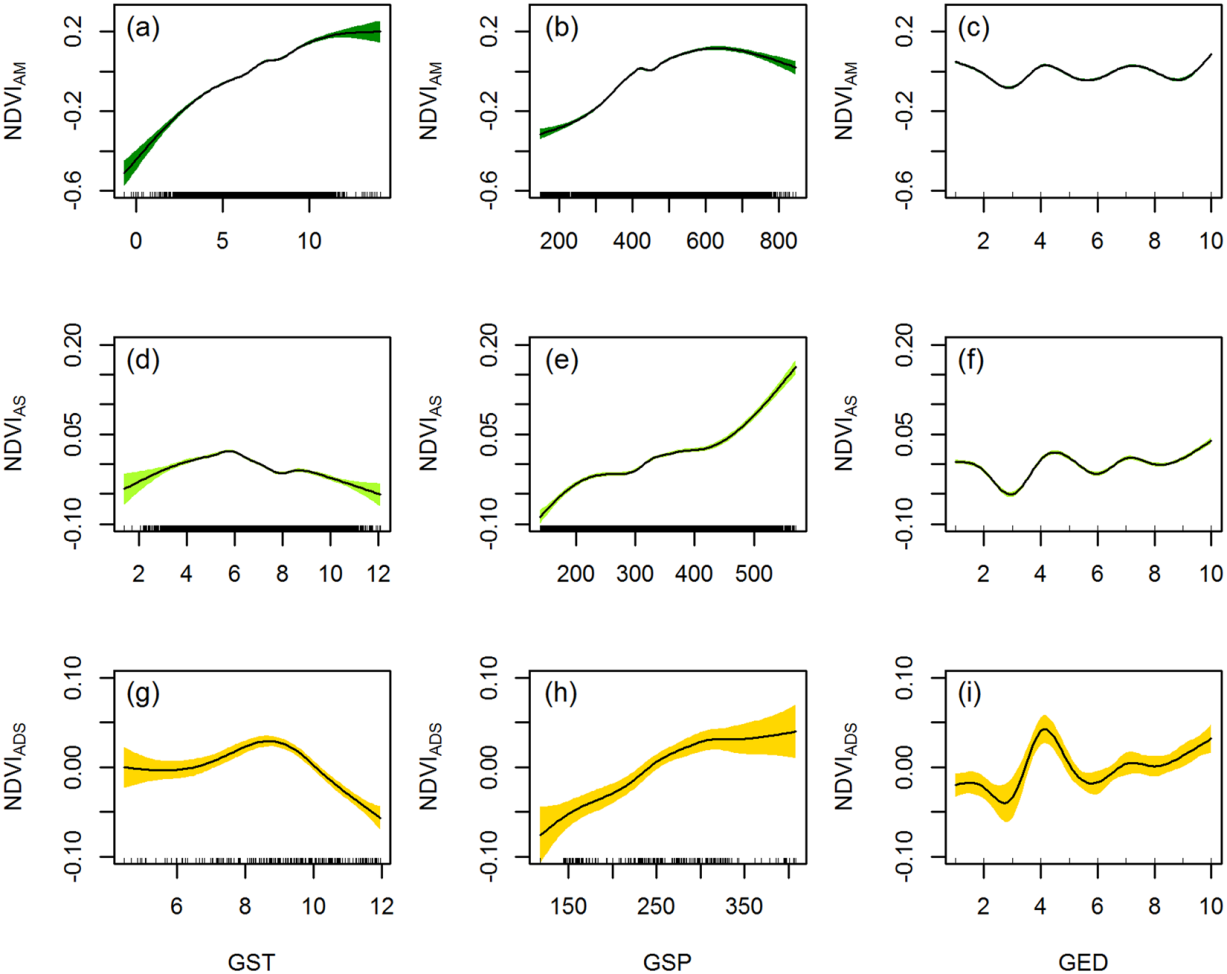
Non-linearity of NDVI over weather conditions and grazing exclusion after fencing. Panels (**a–c**) are for alpine meadows (AM), (**d**–**f**) for alpine steppes, and (**g**–**i**) for alpine desert-steppes. Smoothing curves are estimated from generalized additive models (GAMs) that included growing season temperature (GST), growing season precipitation (GSP), and grazing exclusion duration (GED) as explanatory variables.

Evident non-linearity in changes of NDVI, GST, and GSP over time compared to the corresponding baselines was confirmed by GAMs, with a similar trimodal pattern during the ten-year grazing exclusion (Supplementary Figure S5). However, the magnitude and rhythmicity of the non-linear pattern for each variable differed among grassland types. The GAMs with GST, GSP, and GED as explanatory variables together disentangled their relative influences on the non-linear NDVI restoration (Fig. 3 and Table 2). At the very beginning of GED, until the third year, the NDVI decreased in all types of alpine grasslands. Over the course of the GED, the NDVI reached an equilibrium in meadows, a non-linear increase with a narrowing variability in steppes, and an abrupt increase during the fourth year after fencing followed by a slight, non-linear increase in desert-steppes, respectively (Fig. 3c,f,i, respectively). The interactions between GST and GSP were significant (Supplementary Table S4 and Table S5); however, no clear pattern existed between NDVI and the interaction of GST and GSP (Supplementary Figure S6).

**Table 2:** Summary of generalized additive models (GAMs) that included growing season temperature (GST), growing season precipitation (GSP), and grazing exclusion duration (GED) as explanatory variables for the NDVI variations across fenced grassland patches on the northern Tibetan Plateau.

GAM for	explanatory variable	est. d.f.	est. rank	F	P	adj. R^2^	AIC
alpine meadow (AM)	GST	8.3	8.3	475.2	<2e-16	0.46	−17783.2
	GSP	8.3	9.0	556.1	<2e-16		
	GED	9.0	9.0	144.4	<2e-16		
alpine steppe (AS)	GST	8.5	8.9	77.3	<2e-16	0.26	−28520.9
	GSP	8.1	8.7	171.1	<2e-16		
	GED	9.0	9.0	70.5	<2e-16		
alpine desert-steppe (ADS)	GST	4.5	5.4	37.1	<2e-16	0.60	−924.1
	GSP	4.7	5.7	11.8	7.6e-11		
	GED	8.5	8.9	4.4	3.8e-05		

The relative importance of GST, GSP, and GED was estimated based on the *F*-statistics (Table 2). In alpine meadows, GED had a weaker influence on the NDVI dynamic than GST and GSP across all fenced grassland patches (*F*_GED_: *F*_GST_: *F*_GSP_ = 1: 3.3: 3.9). In alpine steppes, GSP had the strongest influence while GST and GED had comparable influences on the temporal NDVI variation (*F*_GED_: *F*_GST_: *F*_GSP_ = 1: 1.1: 2.4). For alpine desert-steppes, the NDVI was primarily controlled by GST, while GED had the weakest influence on ecosystem self-restoration (*F*_GED_: *F*_GST_: *F*_GSP_ = 1: 2.7: 8.4).

**Figure 4:**
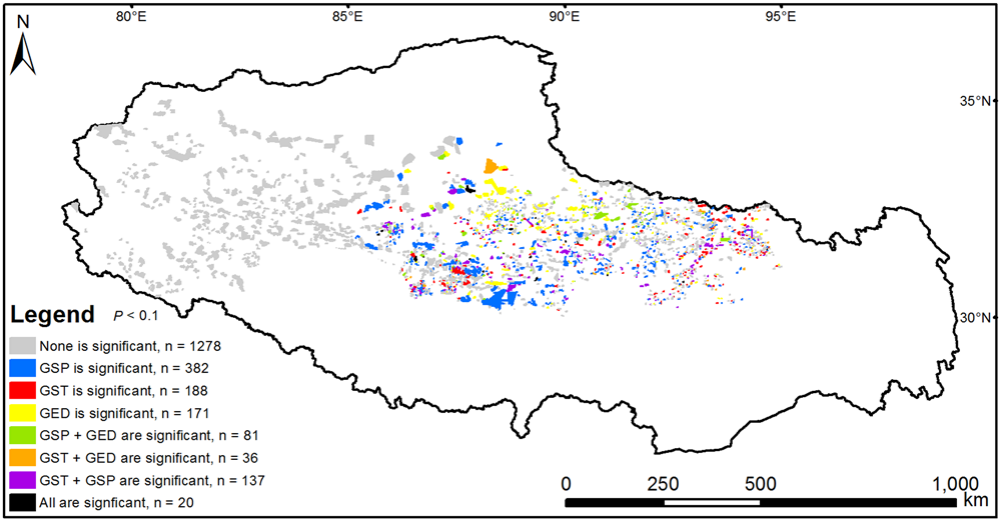
Significance of the effects of weather conditions and grazing exclusion on grassland NDVI changes. The significance was extracted from the generalized additive models (GAMs) that included growing season temperature (GST), growing season precipitation (GSP), and grazing exclusion duration (GED) together as explanatory variables at each fenced grassland patch on the northern Tibetan Plateau. This map was produced in ArcGIS 10.2 (http://www.esri.com).

**Figure 5:**
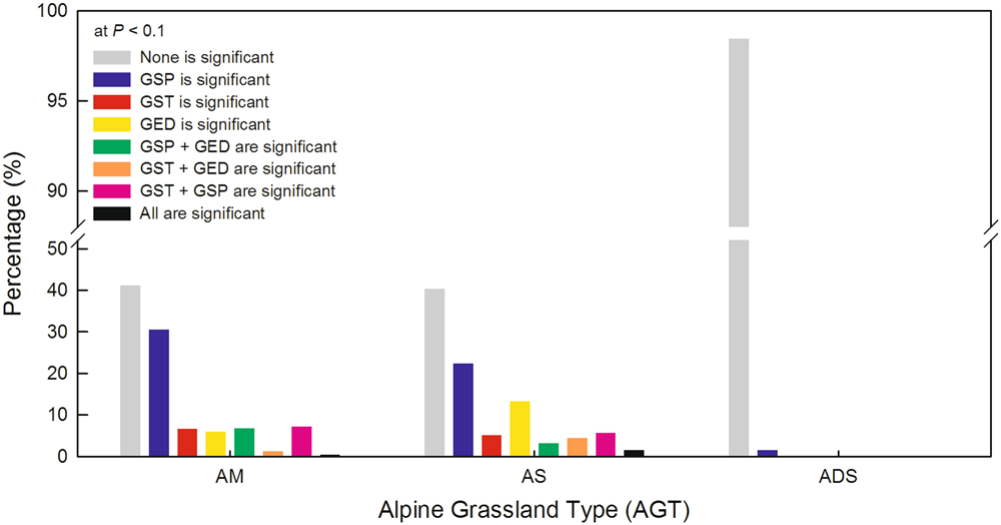
Percentage of fenced patch numbers where grassland NDVI is affected by weather conditions and grazing exclusion, singly or in groups. The significance at *P* < 0.1 was extracted from the generalized additive models (GAMs) that included growing season temperature (GST), growing season precipitation (GSP), and grazing exclusion duration (GED) as explanatory variables at the fenced patch level. AM, AS, and ADS stand for alpine meadow, steppe, and desert-steppe, respectively.

The ten-year GED is likely too short to include explanatory interactions. The GAMs without interactions showed that weather conditions and/or grazing exclusion had a significant influence for each fenced patch only in the eastern region, singly or in groups (Fig. 4, colored patches) but had no influence in the western region (Fig. 4, grey patches). For 41.2% of meadow patches and 40.3% of steppe patches, the NDVI was not affected by neither weather conditions (*P*_GST_ > 0.1 and *P*_GSP_ > 0.1) nor grazing exclusion (*P*_GED_ > 0.1) (Fig. 5 and Supplementary Figure S7). For 1.6% of desert-steppe patches, the NDVI was influenced only by GSP. For only 14.5% of meadow patches and for 22.4% of steppe patches (*P*_GED_ < 0.1) did the GED singly, or together with climatic factors, have significant influences on the NDVI. For 15.6% of meadow patches and for 16.8% of steppe patches (*P* < 0.1), GST had significant influences on the NDVI; whereas for 44.9% of meadow patches and 32.9% of steppe patches (*P* < 0.1), GSP had significant influences on the NDVI (Fig. 5).

## Discussion

Our results revealed the non-linearity of the self-restoration of degraded alpine grassland by grazing exclusion on the northern Tibetan Plateau (Fig. 3). The non-linear NDVI variation likely resulted from the fluctuations of weather conditions over time and across space (Supplementary Figure S5). This variability is partially consistent with the recent finding that aboveground productivity of alpine grasslands non-linearly responds to a regional aridity gradient across the northern Tibetan Plateau^46^.

The increasing of NDVI with temperature in alpine meadows (Fig. 3a) suggests that climate warming would likely benefit meadow plants. The unimodal patterns of NDVI with temperature on steppes and desert-steppes suggest that warming likely limits plant growth in such semi-arid and arid areas (Supplementary Figure S8 and Table S1). This is because drought can reduce plant productivity, limit plant recruitment and settlement, and even affect ecosystem resilience to climate change^47^. Moreover, the unimodal pattern of NDVI with precipitation in alpine meadows also indicates that more precipitation would probably be unutilized and even limit meadow plants’ growth (Fig. 3b and Table 2). Even in the plant growing months, precipitation often falls as snow or hail on alpine meadows; thus, low-temperature stresses co-occur with snow and hail. This pattern might be an explanation for the decline in NDVI with increased precipitation in alpine meadows.

Higher temperature and less precipitation always result in a more serious drought in alpine steppes and desert-steppes (Table 2). The increasing precipitation has a continuously positive influence on steppes but is likely useless to desert-steppes (Fig. 3e and h). This pattern might be due to the differences in community composition and plant properties between steppes and desert-steppes. Plants in alpine desert-steppes that have deeper roots are more tolerant to drought than plants in alpine steppes that have shallow roots^48^. In addition, the small local species pool of alpine desert-steppes^49^ (generally no more than five species per square meter) likely limits its self-restoration because of low species recruitment^24,50^. Thus, the self-restoration of alpine desert-steppes might require a longer grazing exclusion. Moreover, the brief increase in the NDVI in desert-steppes (Fig. 3i) is likely affected by climatic extremes^51,52^. In response to extreme precipitation in the year after drought or in a generally arid area, a rapid demographic increase in dominant grasses can compensate for the loss of dominant forbs and can also result in increases in canopy cover and productivity^53^. These changes might also help explain why neither weather conditions nor grazing exclusion influenced the NDVI in most fenced desert-steppe patches (Figs 4 and 5)

Our results support two of the three hypotheses we proposed to describe the non-linearity and resilience of degraded alpine grasslands after fencing. The non-linearly increasing NDVI with narrowing amplitudes and long-term GED on alpine steppes (Fig. 3f) supports our first hypothesis (**H1**, Fig. 1), and the non-linear equilibrium of NDVI with equable amplitudes over time (Fig. 3a) in alpine meadows supports our second hypothesis (**H2**, Fig. 1). One explanation is that the local species pool of alpine meadows (ranging from 16 to 36 species per square meter) is much bigger than that of alpine steppes (ranging from 6 to 25 species per square meter) and alpine desert-steppes (generally no more than five species per square meter)^49^.

High diversity can stabilize ecosystem functions under human disturbance or climate fluctuations^54,55^. The overlapping niches among species make alpine meadows to be more stable and resistant to perturbations than steppes. This stability is in accordance with recent findings that species-rich plant communities are relatively more resistant to short-term change in management regimes^56,57^. In addition, alpine meadows are generally dominated by *Kobresia* species that can reproduce quickly by cloning. The sparser canopy of alpine steppes relative to that meadows^58^ also suggests much easier recruitment and settlement capabilities for grasses. Thus, the ten-year grazing exclusion by fencing likely promoted the recruitment and settlement of *Stipa* species, and allowed alpine steppe communities to cumulatively benefit from the increasing precipitation. However, we find no evidence to support the third hypothesis (**H3**, Fig. 1) that the grassland degradation rate will be slower due to grazing exclusion by fencing, although the NDVI declined in some patches compared to the baseline before fencing (Fig. 2a). This result implies that grazing exclusion at least has no apparent adverse influence on alpine grassland restoration.

The GAMs in Fig. 3 combined both temporal and spatial variability simultaneously. Additionally, we mapped the significance of each explanatory variable at the fenced patch scale (Fig. 4), with eight different scenarios where weather conditions and grazing exclusion affected NDVI, singly or in groups. Neither weather conditions nor grazing exclusion had a significant influence on the NDVI for 1278 desert-steppe patches where the GSP is generally less than 300 mm (Supplementary Figure S8d). The NDVI of fenced meadow and steppe patches was likely affected by weather conditions or grazing exclusion (Fig. 4). GSP and GST significantly affected the NDVI (*P* < 0.1) at 630 and 381 patches, respectively, which accounted for 27.5% and 16.6% of all fenced patches (Figs 4 and 5). GED significantly affected the NDVI only at 308 patches, which accounted for 13.4% of all fenced grassland patches ((Figs 4 and 5).

The proportion of fenced patches where weather conditions and grazing exclusion significantly affected the NDVI was somewhat low; however, a more detailed field observation of compositional changes is necessary to examine the effects of fencing on the restoration of degraded grasslands as the prediction of ecosystem resilience following disturbance is dependent on which components of the ecosystem are investigated^59^. The certain proportion of fenced grasslands where neither weather conditions nor grazing exclusion affected the NDVI over time might indicate that there was no serious degradation before fencing (Supplementary Figure S9).

Both ecosystem degradation and restoration are not necessarily expected to be linear under climate change and human disturbance. In a Mediterranean rangeland, Saatkamp, *et al*.^60^ found that plant functional traits have a non-linear manner in response to grazing intensity. On the northern Tibetan Plateau, Wu, *et al*.^46^ also confirmed that plant functional trait diversity can regulate the non-linear pattern of community productivity along the regional climatic gradients. Therefore, a scientific assessment of grazing exclusion effectiveness requires more evidence from detailed field observations and a better knowledge of the mechanisms of the relationship between biodiversity and ecosystem functionality. Nevertheless, our results demonstrate that time series of remote sensing data can be used to map the non-linearity of ecological processes and the resilience of ecosystems recovering from perturbation or degradation.

## Materials and Methods

### Study area

This study was conducted on the northern Tibetan Plateau, which is the most important area for the Tibetan herding families^28,61^. The plateau experiences a continental monsoon climate, with mean annual temperature (MAT) ranging from −2.3 °C to 1.2 °C and mean annual precipitation (MAP) from less than 100 mm to more than 450 mm^49^. Up to 85% of the total precipitation occurs from May to September, when the daily air temperature is higher than 5 °C^49^ (Supplementary Figure S8). In this study, fenced grassland patches range in GSP from 193 mm to 485 mm, and in GST from 0 °C to 13.8 °C (Supplementary Table 1). The climate-vegetation zones encountered, from east to west, are an alpine semi-humid zone where *Kobresia* meadow dominates to an alpine semi-arid zone where *Stipa* steppe is distributed and finally to an alpine arid zone where *Stipa* desert-steppe is found^3^. Alpine grasslands on the northern Tibetan Plateau are grazed by domestic livestock (yaks, sheep, and goats) and wild herbivores (Tibetan antelopes and kiangs).

### Data collection

Weather conditions from May to September are critical for plant growth in alpine grasslands^62–65^, and this period is generally accepted as the plant growing season on the Tibetan Plateau. Temperature and precipitation records during the plant growing season of 2001–2015 were downloaded from the Meteorology Information Center of the Chinese National Bureau of Meteorology (http://data.cma.cn). Weather records of the 200 observation stations located within and around the Qinghai-Tibetan Plateau^17^ were interpolated into raster surfaces with a 1 km spatial resolution by using ANUSPLIN 4.3^66^. Subsequently, raster surfaces of temperature and precipitation were averaged to a time scale of the plant growing season and then extracted to each fenced patch in ArcGIS 10.2.

NDVI is the most frequently used proxy for quantifying productivity, aboveground biomass, and vegetation cover of diverse ecosystems^67^. The monthly MOD13A3 NDVIs from May to September with a 1 km spatial resolution were used in this study. All NDVI data were calibrated for errors caused by adverse atmospheric, radiometric, and geometric conditions using TIMESAT 2.3^68^. The records of fenced grassland patch locations, including the longitude, altitude, and elevation of the vertices of each fenced patch (polygon), were provided by the agriculture and husbandry bureau of each county. We shaped patch location records into polygons in ArcGIS 10.2. Because the patch size varied from 49 to 157,343 hectares, to avoid potential influences of outside pixels where grasslands are under grazing, patches smaller than 100 hectares were excluded from this study (Figure S1). Finally, growing season NDVIs were averaged and extracted to each fenced patch in ArcGIS 10.2.

### Data analysis

Using GAMs^69^, we described the relationships of the NDVI with weather variables and grazing exclusion across three alpine grassland types on the northern Tibetan Plateau. GAMs are a flexible nonparametric tool to detect the non-linearity of relationships between the response and the predictors^70^. They assume that functions are additive and that components are smooth. Therefore, we would find solid evidence to support our alternative hypotheses if we found a non-linear relationship of NDVI increasing, maintaining equilibrium, or decreasing in response to grazing exclusion.

For stage one, we first divided the whole study period into two subperiods of before (2001–2005) and since being fenced (2006–2015). Subsequently, we compared the averaged NDVIs between the two sub-periods to detect the direction in which degraded grassland patches are generally trending, increasing, decreasing, or no change. For stage two, we calculated the difference of yearly values during the second sub-period for NDVI, GST, and GED relative to the averages of the first sub-period. Thus, ΔNDVIs were analyzed in a GAM using the *mgcv* package to show its non-linear responses to ΔGST, ΔGSP, and GED. In addition to the non-linearity, the temporal tendency of ΔNDVI, ΔGST, and ΔGSP was also determined with simple linear regressions. For each patch, the ten-year fencing is likely too short to obtain a robust regression analysis of NDVI with GST, GSP, or GED. Instead, we ran correlation analyses of NDVI with GST and GSP and mapped the correlation coefficient and the corresponding significance for each fenced patch.

The relative importance of weather conditions and grazing exclusion was estimated by comparing the *F*-statistic between explanatory variables in GAMs. To disentangle the relative importance of GST, GSP, and GED, as well as their interactions, GLMs were additively used for the yearly NDVIs of the second sub-period, using the *nlme* package (Supplementary Table S4), because a ten-year GED is likely too short and GAMs across patches did not indicate evident patterns of the NDVI in response to the GST × GSP interactions (Supplementary Figure S9). In this study, we ran GAMs without interactions again for each fenced patch, extracted the explanatory variable’s significance values from model summary tables, and mapped them with the corresponding information of alpine grassland types. As each explanatory variable might be significant or not, therefore, eight different scenarios were generated, with GST, GSP, and GED singly or in groups affecting the NDVI. Finally, we calculated the percentage of patch numbers and area of each scenario accounting for all fenced patches within each alpine grassland type.

All maps were produced in ArcGIS 10.2, bar graphs in SigmaPlot 12.5, and others in RStudio.

## Electronic supplementary material

Supplementary file
