# Supplementary material for: Grazing exclusion by fencing non-linearly restored the degraded alpine grasslands on the Tibetan Plateau
Source: Sci Rep. 2017 Nov 9;7:15202. doi: 10.1038/s41598-017-15530-2 (PMC5680212; doi:10.1038/s41598-017-15530-2)
Supplement: Supplementary file 1 — Supplementary file [file 41598_2017_15530_MOESM1_ESM.pdf]

Grazing exclusion by fencing non-linearly restored degraded alpine grasslands on the Tibetan Plateau

Jianshuang Wu[1*,2,3], Yunfei Feng[1], Xianzhou Zhang[1*], Susanne Wurst[2], Britta Tietjen[3], Paolo Tarolli[4], Chunqiao Song[5]

Resilience is an important aspect of the non-linear restoration of disturbed ecosystems. Fenced grassland patches on the northern Tibetan Plateau can be used to examine the resistance and resilience of degraded alpine grasslands to grazing and to a changing climate. To examine the non-linearity of restoration, we used moderate resolution imaging spectroradiometer (MODIS) normalized difference vegetation index (NDVI) as a proxy for productivity during a ten-year restoration by fencing. Degraded alpine grasslands exhibited three restoration trajectories: an equilibrium in meadows, a non-linear increase across steppes, and an abrupt impulse in desert-steppes following a slight increase in productivity. Combined with weather conditions, the ten-year grazing exclusion has successfully enhanced the NDVI on the most degraded steppes, but did not do so efficiently on either meadows or desert-steppes. Warming favors the NDVI enhancement of degraded meadows, but higher temperatures limited the restoration of degraded steppes and desert-steppes. Precipitation is necessary to restore degraded alpine grasslands, but more precipitation might be useless for meadows due to lower temperatures and for desert-steppes due to limitations caused by the small species pool. We suggest that detailed field observations of community compositional changes are necessary to better understand the mechanisms behind such non-linear ecological restorations.

[1] Lhasa Plateau Ecosystem Research Station, Key Laboratory of Ecosystem Network Observation and Modelling, Institute of Geographic Sciences and Natural Resources Research, Chinese Academy of Sciences, Beijing 100101, China. [2] Functional Biodiversity, Institute of Biology, Free University of Berlin, 14195 Berlin, Germany. [3] Biodiversity-Ecological Modelling, Institute of Biology, Free University of Berlin, 14195 Berlin, Germany. [4] Department of Land, Environment, Agriculture and Forestry, University of Padova, Agripolis, viale dell'Università, 16, Legnaro (PD), Italy. [5] Department of Geography, University of California, Los Angeles, CA 90095, USA. Correspondence and requests for materials should be addressed to J. Wu (wujs.07s@igsnrr.ac.cn) or X. Zhang (zhangxz@igsnrr.ac.cn).

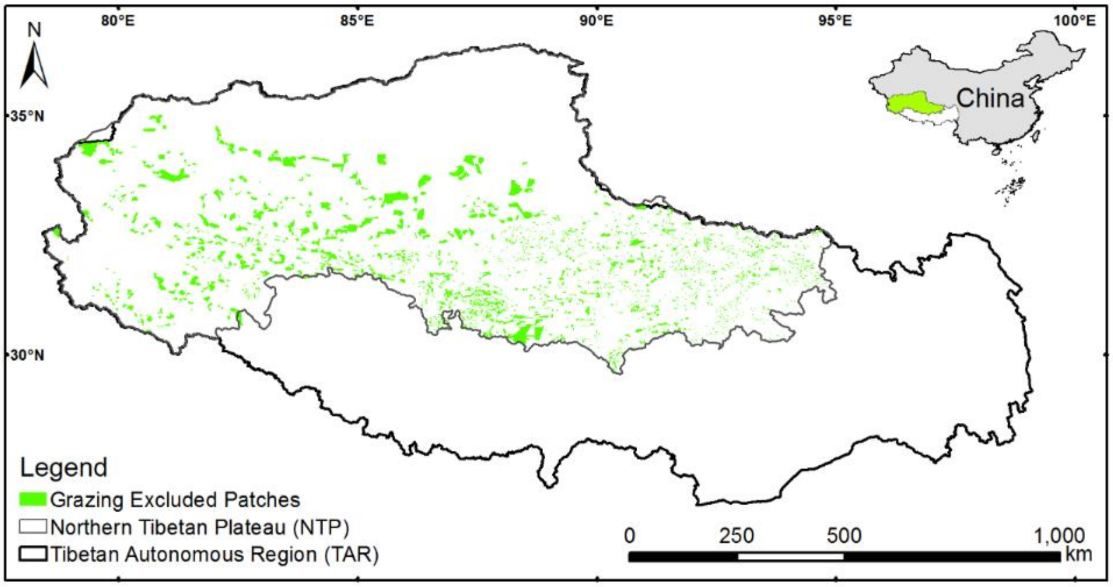

Figure S1. Spatial distribution of the fenced patches in degraded alpine grasslands on the northern Tibetan Plateau. This map was produced in ArcGIS 10.2 (http://www.esri.com). The geographic coordinates including the longitude, altitude, and elevation of the vertices of each fenced grassland patch (polygon) were provided by the agriculture and husbandry bureau of each county in the study area.

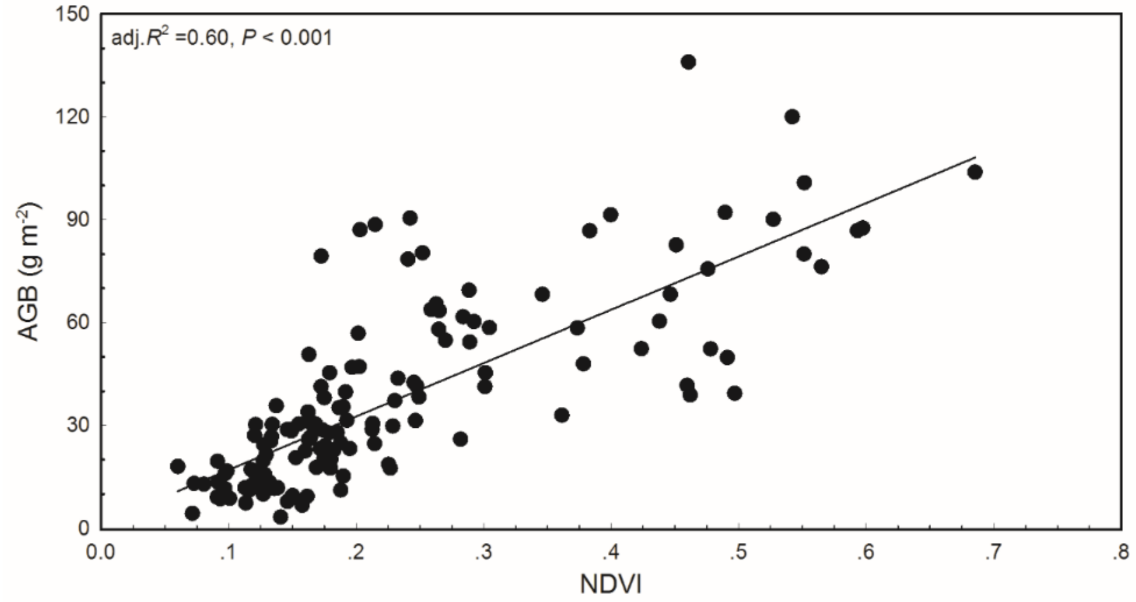

Figure S2. The relationship between Normalized Difference Vegetation Index (NDVI) and aboveground biomass (AGB) on the Northern Tibetan Plateau. AGB data were cited from the references 1-3.

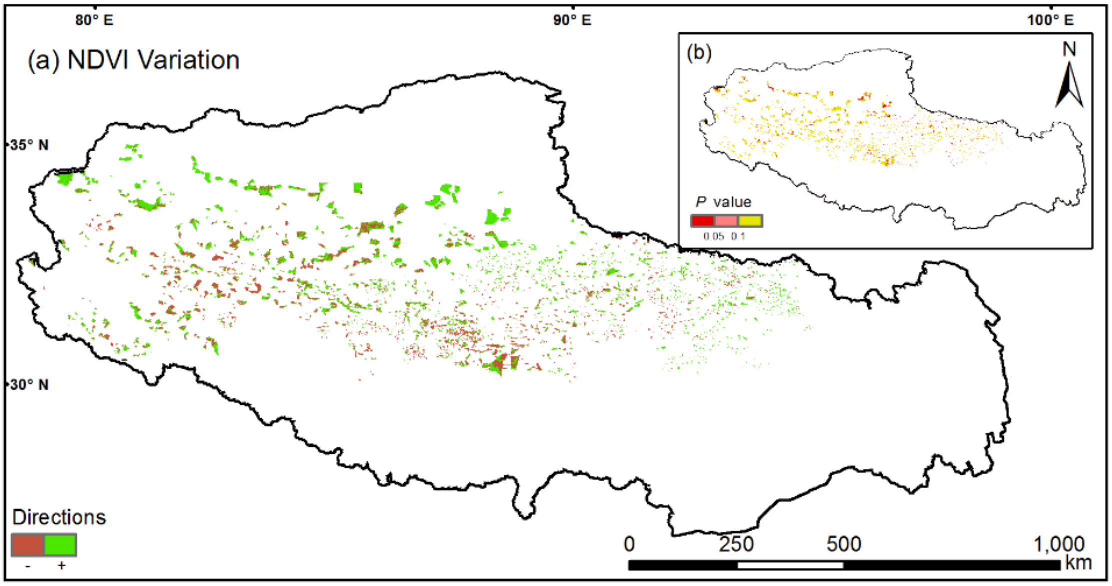

Figure S3. Normalized Difference Vegetation Index (NDVI) variation at the grassland patches fenced from 2006 to 2015. In panel (a), the directions were estimated from the slopes of linear regressions, and green and red colors indicate increasing and decreasing, respectively. In panel (b), the significance of the regression slopes at $P < 0.05$, $P < 0.1$, and $P > 0.1$ was shown in red, pink, and yellow, respectively. This map was produced in ArcGIS 10.2 (http://www.esri.com).

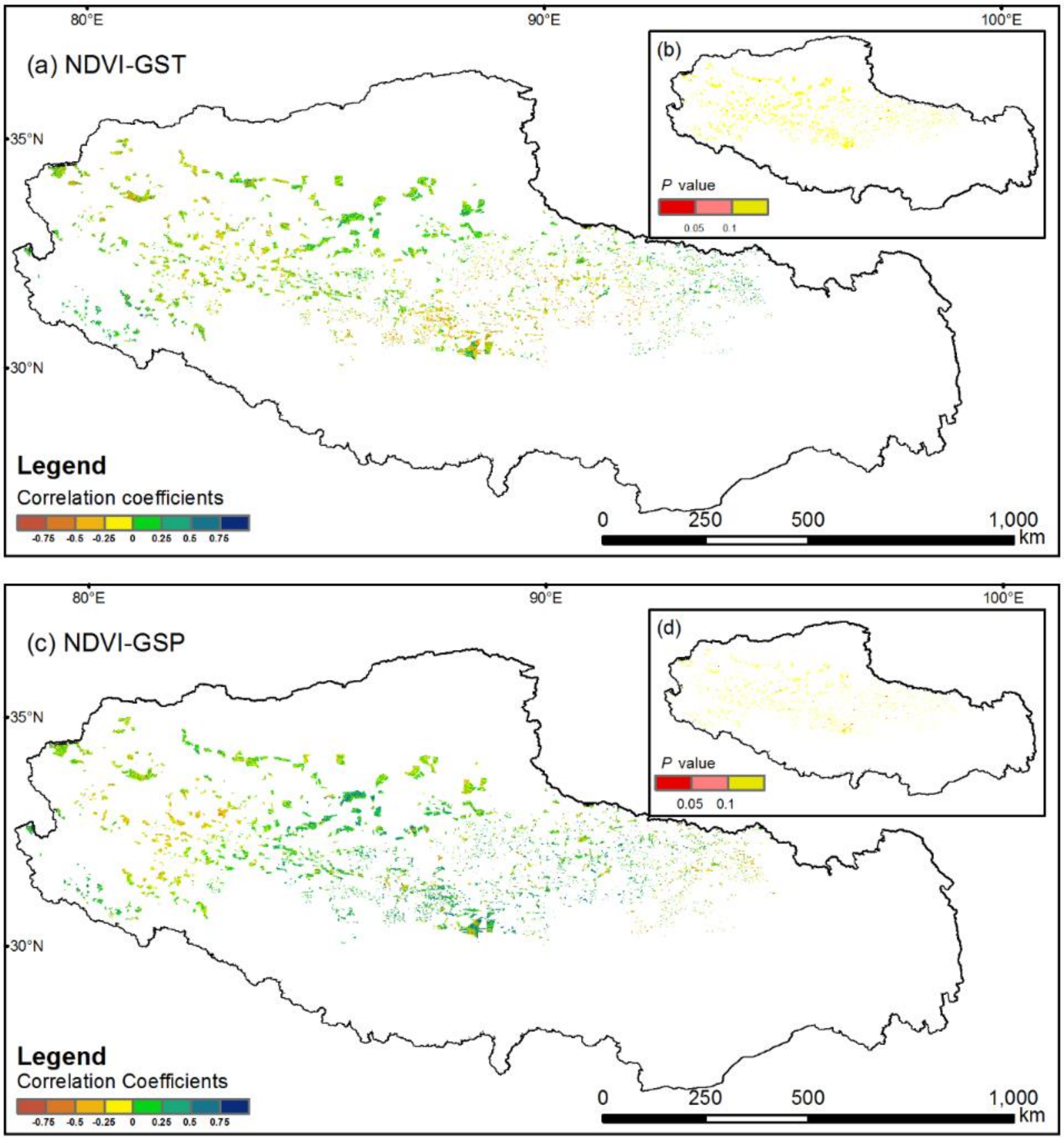

Figure S4. Correlation coefficients (panels a & c) and the corresponding significance (panels b & d) of the Normalized Difference Vegetation Index (NDVI) with growing season temperature (GST, panels a & b) and growing season precipitation (GSP, panels c & d) at the grassland patches fenced from 2006 to 2015 on the northern Tibetan Plateau. The two maps were produced in ArcGIS 10.2 (http://www.esri.com).

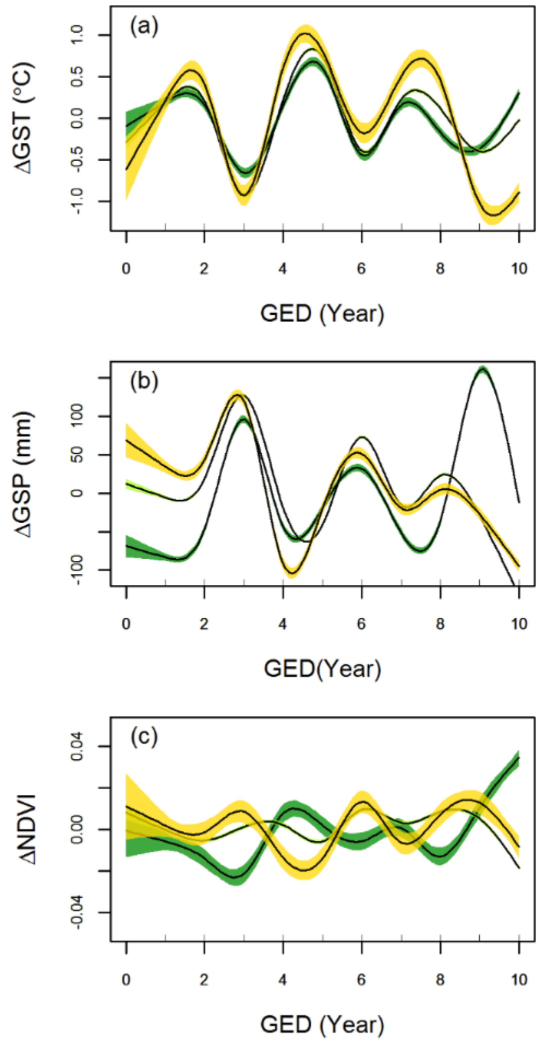

Figure S5. Non-linear patterns of (a) growing season temperature (GST), (b) growing season precipitation (GSP), and (c) Normalized Difference Vegetation Index (NDVI) across the fenced grassland patches within each alpine grassland type. Differences of annual values of 2006-2015 for each variable relative to the baselines, the averages of 2001-2005 at each patch, were analyzed in generalized additive models (GAMs) that only included grazing exclusion duration (GED) as the explanatory variable. Dark green lines for alpine meadows, green lines for steppes, and yellow lines for desert-steppes.

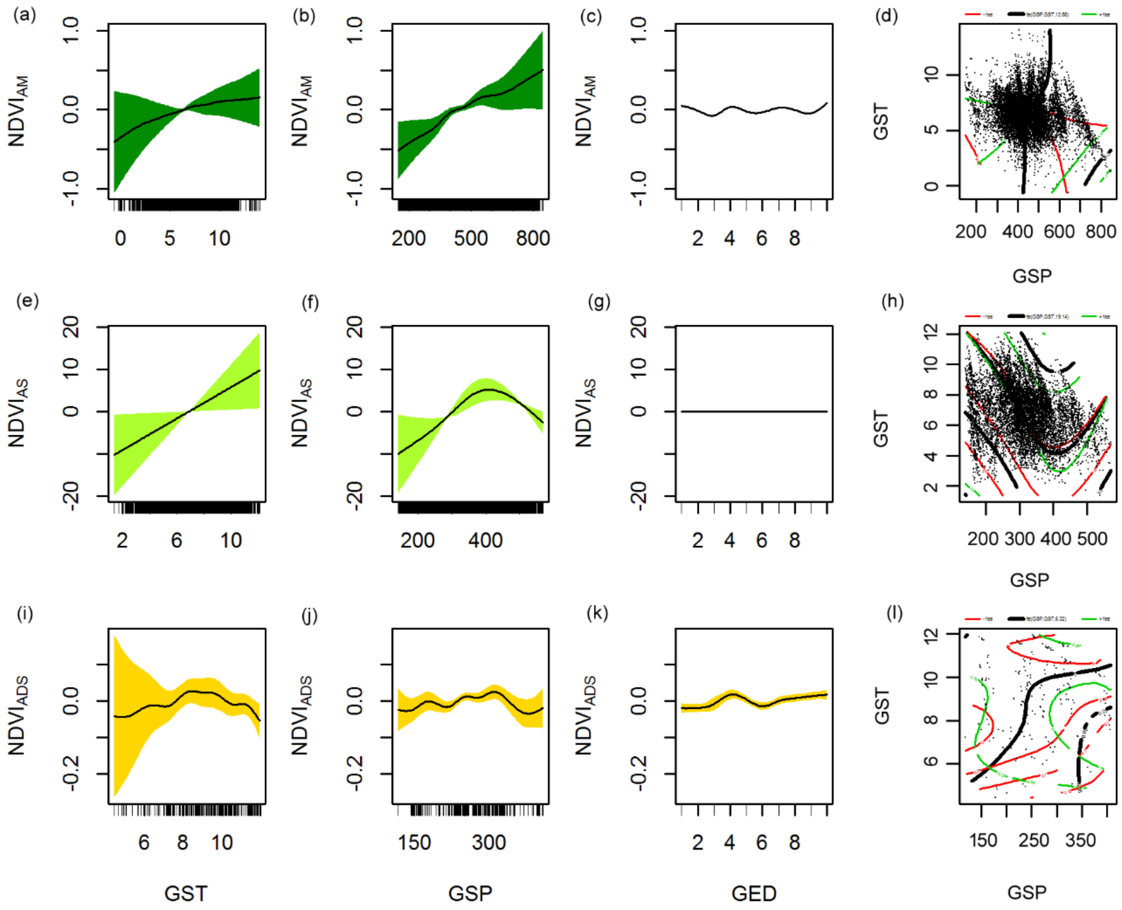

Figure S6. Non-linearity of the NDVI after fencing over weather conditions and grazing exclusion. Panels (a)-(d) are for alpine meadows (AM), (e)-(h) for alpine steppes, and (i)-(l) for alpine desert-steppes. Smoothing curves are estimated from generalized additive models (GAMs) that included growing season temperature (GST), growing season precipitation (GSP), grazing exclusion duration (GED), and the interaction of GSP*GST together as explanatory variables. Panels (d), (h), and (l) are for the effects of GSP*GST interaction on the NDVI variation in AM, AS, and ADS, respectively. Red, black, green lines in panels (d), (h), and (l) stand for -1 standardized error, estimated smoothing contours for NDVI, and +1 standardized error, respectively.

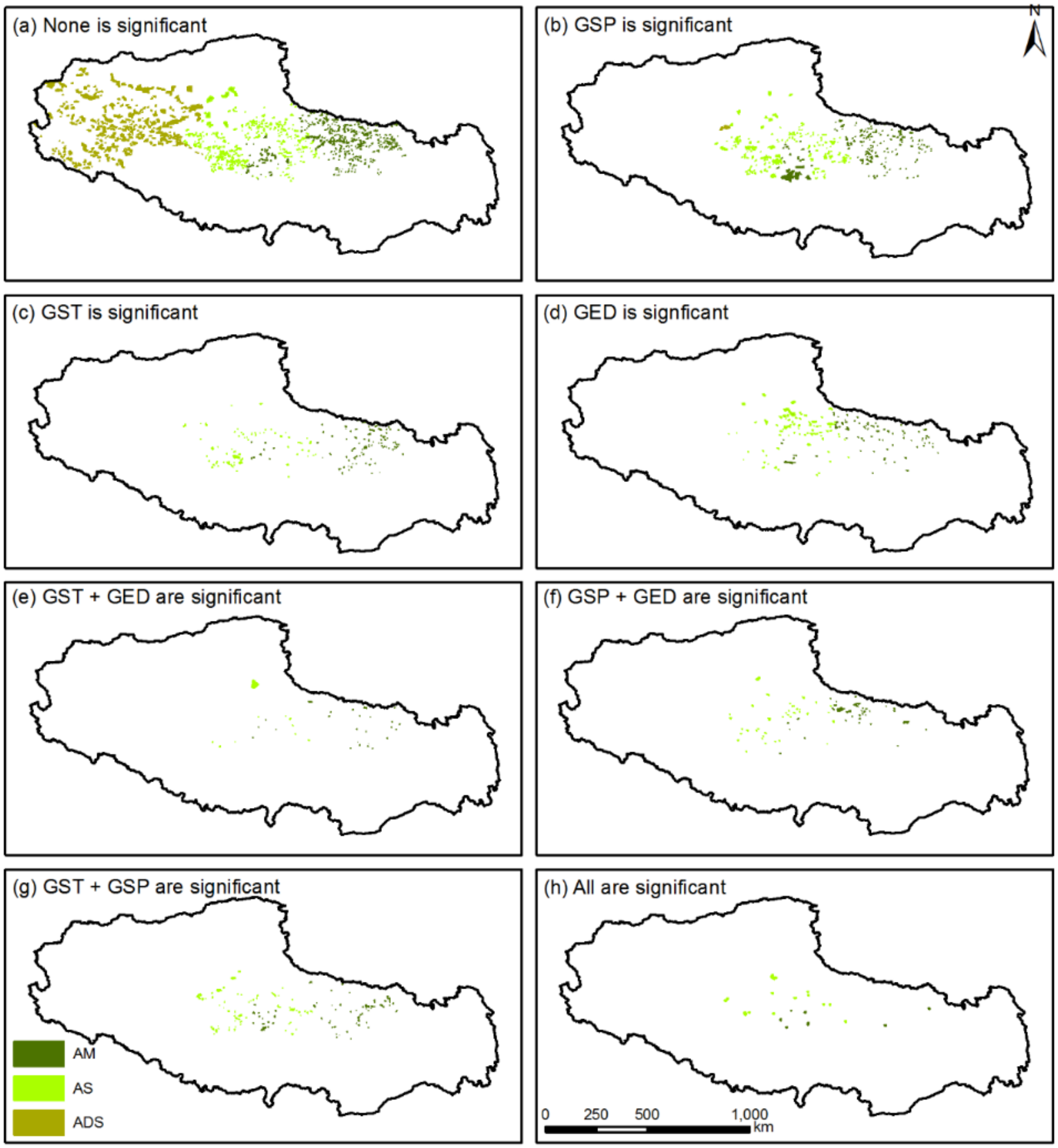

Figure S7. Spatial distribution of fenced grassland patches where growing season temperature (GST), growing season precipitation (GSP), and grazing exclusion duration (GED) were singly or in groups to drive the temporal Normalized Difference Vegetation Index (NDVI) dynamic at $P < 0.1$ level within alpine meadow (AM), alpine steppe (AS) and alpine desert-steppe (ADS), respectively, on the northern Tibetan Plateau. The significance was extracted from the generalized additive model (GAMs) at the patch level that included GST, GSP, and GED as explanatory variables. These maps were produced in ArcGIS 10.2 (http://www.esri.com).

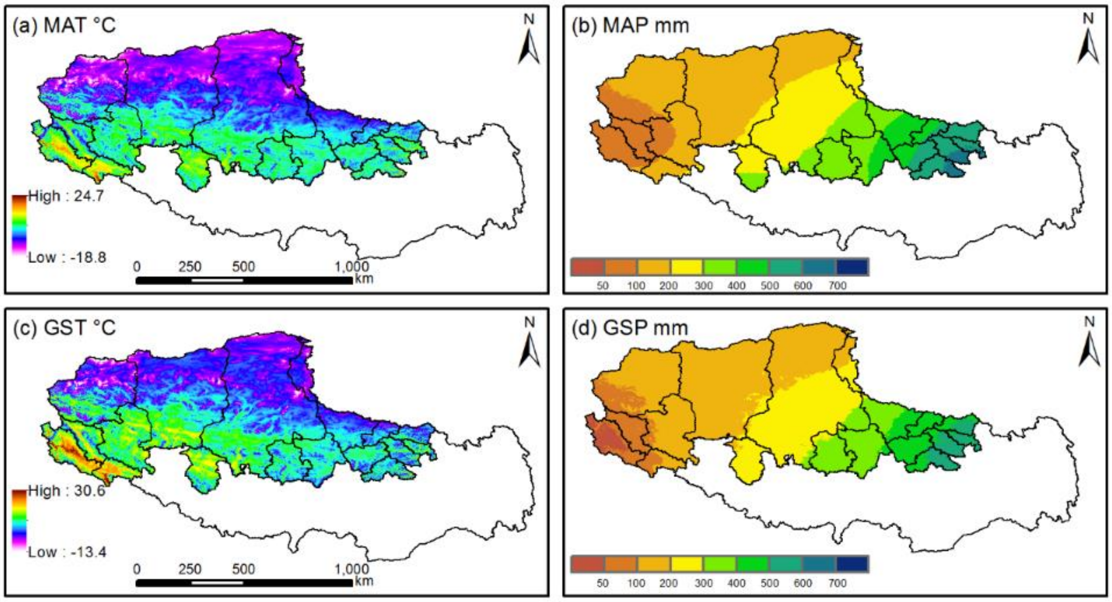

Figure S8. Climatic gradients based on the mean annual values of 1986-2015 across the northern Tibetan Plateau. (a) MAT, mean annual temperature; (b) MAP, mean annual precipitation; (c) GST, growing season temperature; (d) GSP, growing season precipitation. These maps were produced in ArcGIS 10.2 (http://www.esri.com).

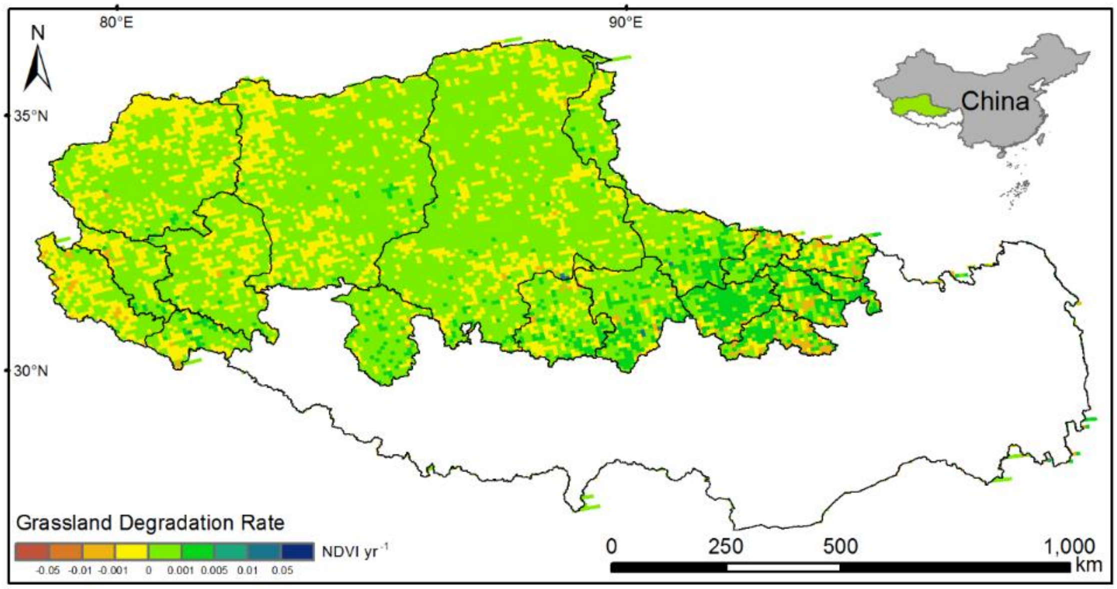

Figure S9. Spatial pattern of grassland degradation trends on the northern Tibetan Plateau. The trend was estimated from the slope of linear regression using the yearly maximum NDVI from 1986 to 2005. This map was produced in ArcGIS 10.2 (http://www.esri.com).

Table S1. Numbers, size range, total area, and climate conditions of the fenced grassland patches within different alpine grassland types on the northern Tibetan Plateau. From May to September was determined as the plant growing season. GST and GSP are for growing season temperature and growing season precipitation, respectively. Fenced patches smaller than 100 hectares were excluded from this study.

	numbers	size range (hectare)	total area (hectare)	GST range (°C)	GSP range (mm)
alpine meadow (AM)	1214	56~157343	972352	0~13.8	332.4~485.9
alpine steppe (AS)	1038	74~97563	1483286	2.4~11.5	248.8~366.5
alpine desert-steppe (ADS)	221	49~96021	2300873	6.2~11.4	193.0~309.3

Table S2. Significant increasing and decreasing trends of the NDVI at grassland patches fenced from 2006 to 2015 were summarized at the 0.05 and 0.1 levels from linear regressions within alpine meadow (AM), alpine steppe (AS), alpine desert steppe (ADS), respectively, and across the northern Tibetan Plateau.

	increasing		decreasing	
	at $P < 0.05$ level (%)	at $P < 0.1$ level (%)	at $P < 0.05$ level (%)	at $P < 0.1$ level (%)
alpine meadow (AM)	5.77	11.7	3.71	6.59
alpine steppe (AS)	3.28	5.68	1.25	3.28
alpine desert-steppe (ADS)	0.9	1.36	0.9	0.9
total	2.14	4.12	1.21	2.35

Table S3. Correlations of the NDVI at the fenced grassland patches with growing season temperature and precipitation (GST and GSP) from 2006 to 2015 within alpine meadow (AM), steppe (AS), desert steppe (ADS), respectively, across the northern Tibetan Plateau.

	significantly only with GST %	significantly only with GSP %	significantly with both GST and GSP %	non-significantly with neither GST nor GSP %
alpine meadow (AM)	1.82	1.90	0.74	95.54
alpine steppe (AS)	0.48	2.51	0.19	96.81
alpine desert-steppe (ADS)	0.00	0.46	0.00	99.54
total	1.09	2.03	0.45	96.43

Table S4. Summary of generalized linear models (GLMs) with growing season temperature (GST), growing season precipitation (GSP), and grazing exclusion duration (GED) together as explanatory variables for the NDVI dynamics across fenced grassland patches within each alpine grassland type.

	alpine meadow AIC = -16350.9		alpine steppe AIC = -27408.1		alpine desert-steppe AIC = -805.9	
	t	*P*	t	*P*	t	*P*
Intercept	7.0	2.7e-12	7.8	7.2e-15	1.4	0.17
GST	-4.9	**8.5e-07**	-1.6	0.11	-0.3	0.74
GSP	-7.0	**3.4e-12**	1.0	0.34	0.2	0.83
GED	1.2	0.22	-2.99	**< 0.01**	-1.9	**0.06**
GST * GSP	11.5	2e-16	0.4	0.71	0.1	0.92
GST * GED	-1.7	**0.09**	1.9	**0.06**	1.8	**0.07**
GSP * GED	0.9	0.37	5.1	**4e-07**	2.3	**< 0.05**
GST * GSP * GED	-0.4	0.72	-3.2	**< 0.01**	-2.1	**< 0.05**

Table S5. Summary of generalized additive models (GAMs) that included growing season temperature (GST), growing season precipitation (GSP), and grazing exclusion duration (GED) as explanatory variables for the NDVI variations across the fenced grassland patches on the northern Tibetan Plateau.

GAM for	explanators	est. *d.f.*	est. rank	*F*	*P*	adj.R^2	AIC
alpine meadow (AM)	GST	7.3	7.9	3.1	0.002	0.49	-18409.5
	GSP	8.4	8.6	30.5	<2e-16		
	GED	9.0	9.0	133.1	<2e-16		
	GSP*GST	12.9	14.4	34.1	<2e-16		
alpine steppe (AS)	GST	7.1	7.6	9.2	3.6e-12	0.28	-28740.3
	GSP	8.9	9.0	22.9	<2e-16		
	GED	8.9	9.0	67.8	<2e-16		
	GSP*GST	19.1	19.7	12.5	<2e-16		
alpine desert-steppe (ADS)	GST	8.6	8.8	2.4	0.012	0.70	-969.9
	GSP	7.9	8.6	2.9	0.002		
	GED	7.2	8.1	2.7	0.008		
	GSP*GST	6.3	7.9	2.7	0.014		

References

1 Wu, J. S. *et al.* Effects of livestock exclusion and climate change on aboveground biomass accumulation in alpine pastures across the Northern Tibetan Plateau. *Chinese Sci Bull* **59**, 4332-4340 (2014).

2 Zeng, C. X., Wu, J. S. & Zhang, X. Z. Effects of Grazing on Above- vs. Below-Ground Biomass Allocation of Alpine Grasslands on the Northern Tibetan Plateau. *Plos One* **10**, doi:10.1371/journal.pone.0135173 (2015).

3 Wu, J. S. *et al.* Grazing-Exclusion Effects on Aboveground Biomass and Water-Use Efficiency of Alpine Grasslands on the Northern Tibetan Plateau. *Rangeland Ecol Manag* **66**, 454-461 (2013).
